# Supplementary figures and images for: Lipidomic and Ultrastructural Characterization of the Cell Envelope of Staphylococcus aureus Grown in the Presence of Human Serum
Source: mSphere. 2020 Jun 17;5(3):e00339-20. doi: 10.1128/mSphere.00339-20 (PMC7300354; doi:10.1128/mSphere.00339-20)

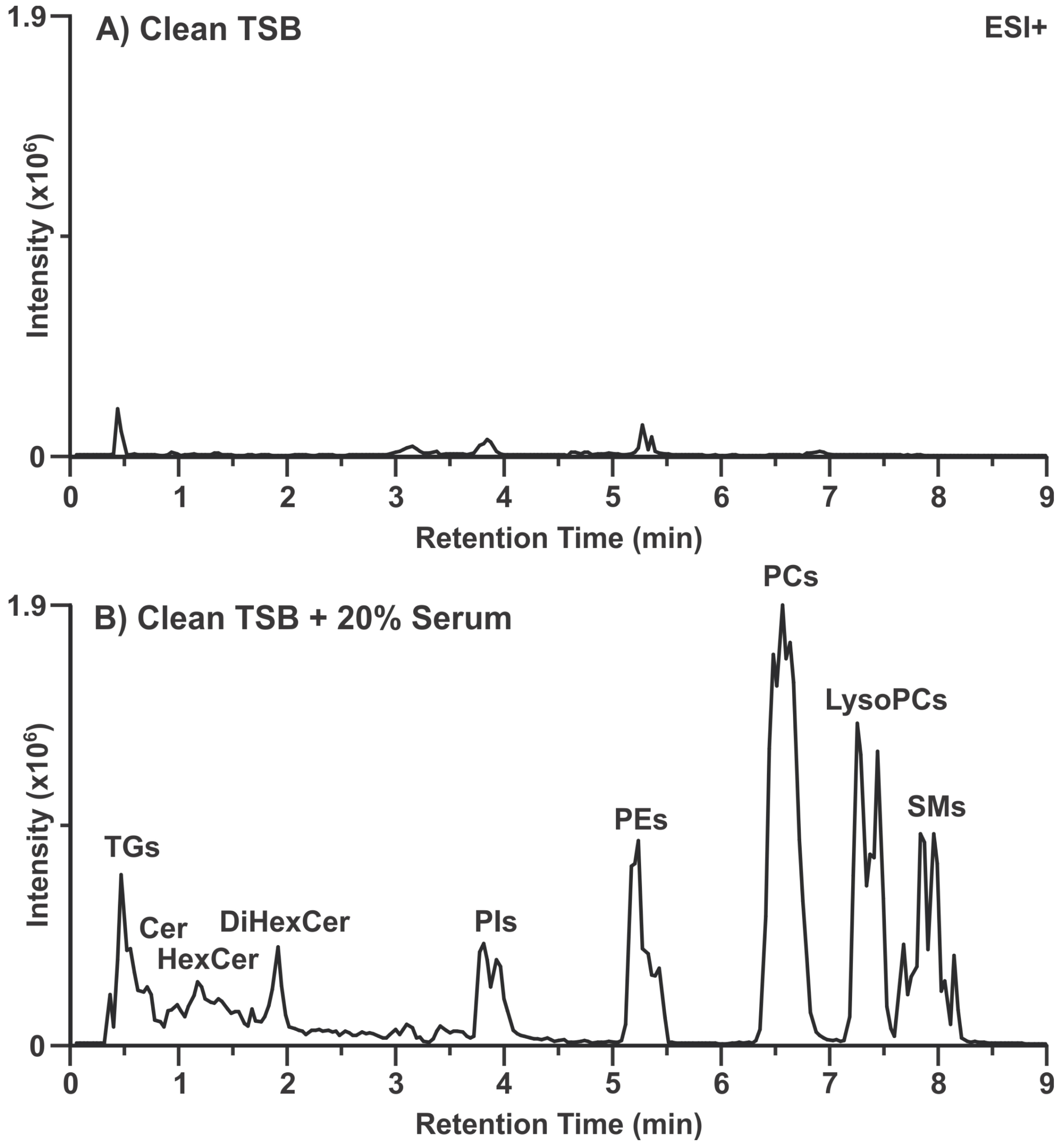

Supplement: FIG S1 [file mSphere.00339-20-sf001.jpg]

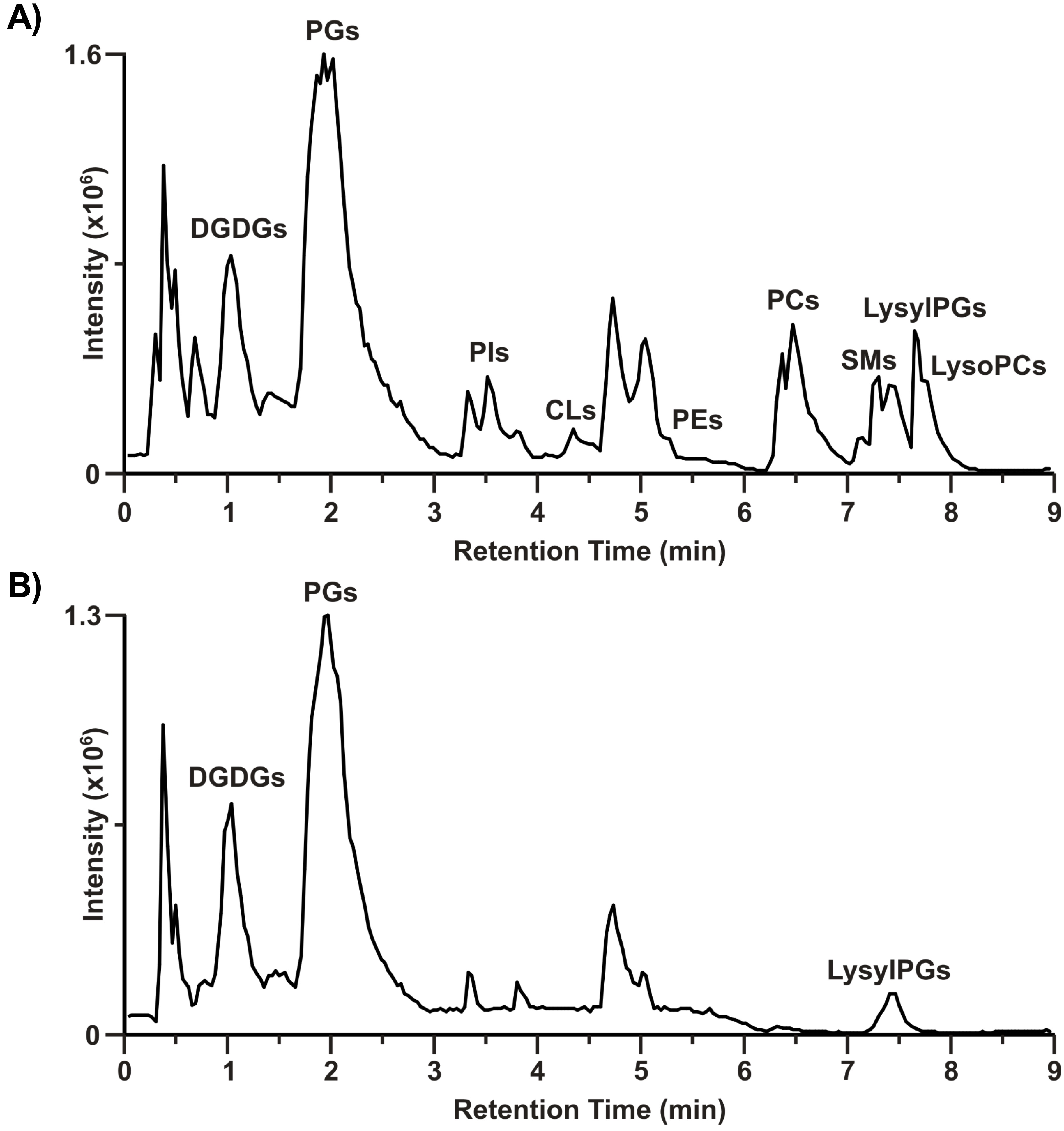

Supplement: FIG S2 [file mSphere.00339-20-sf002.jpg]

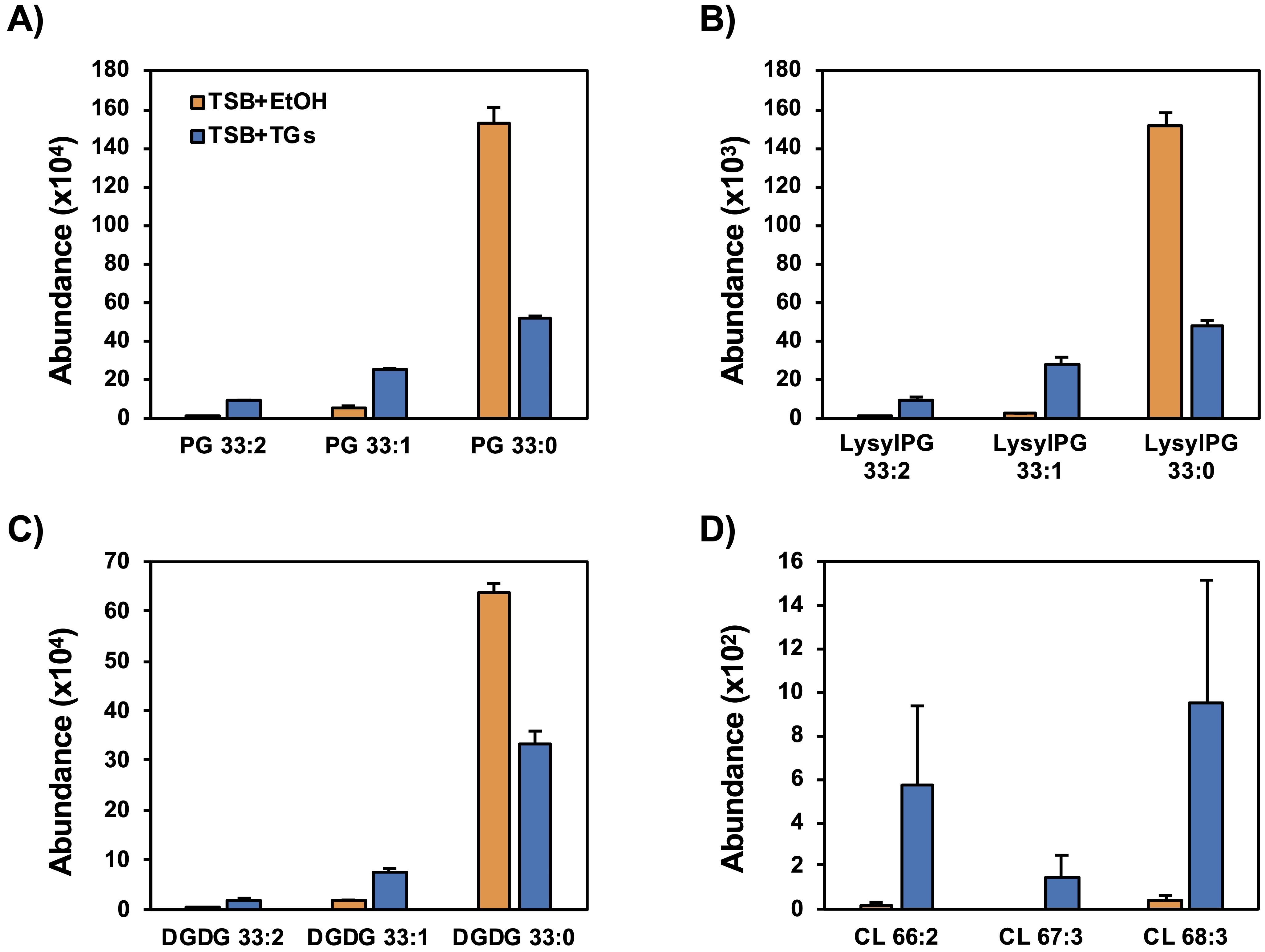

Supplement: FIG S3 [file mSphere.00339-20-sf003.jpg]

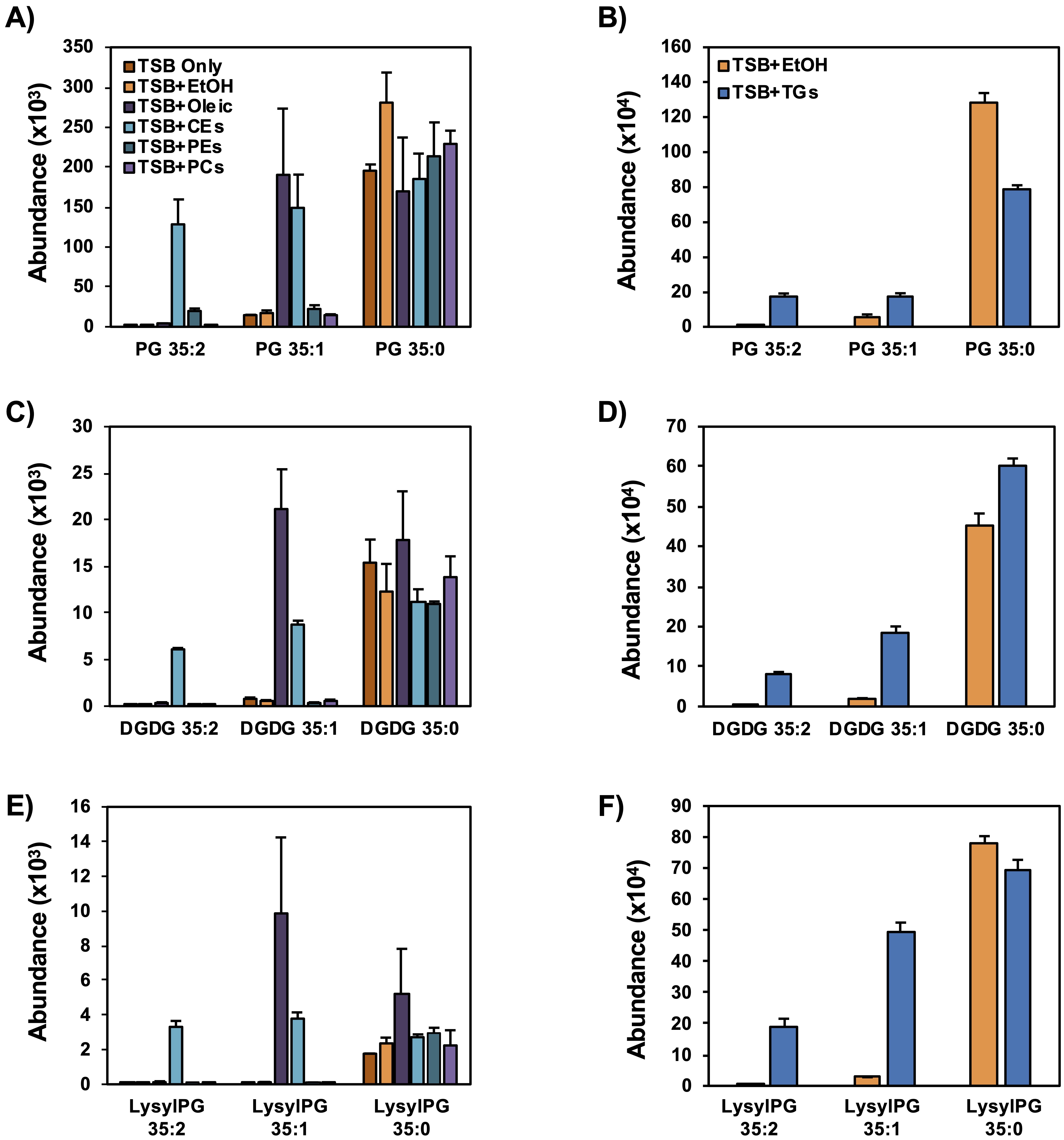

Supplement: FIG S4 [file mSphere.00339-20-sf004.jpg]
